# Left Ventricular Remodeling After Total Coronary Revascularization via Anterior Thoracotomy Versus Conventional Coronary Artery Bypass Grafting

**DOI:** 10.3390/jcdd13060244

**Published:** 2026-06-03

**Authors:** Vedat Aslan, Sefa Sural, Özerdem Özçalışkan, Gökhan Gökaslan

**Affiliations:** 1 Vocational School of Health Services, Toros University, Mersin 33140, Türkiye; drsefasural@yahoo.com; 2 Department of Cardiovascular Surgery, Medicalpark Hospital, Mersin 33200, Türkiye; ozerdemo@yahoo.com; 3 Department of Cardiovascular Surgery, Gaziantep Anka Hospital, Gaziantep 27590, Türkiye; cerrah06@yahoo.com

**Keywords:** total coronary revascularization via anterior thoracotomy, coronary artery bypass grafting, left ventricular ejection fraction, minimally invasive cardiac surgery, ventricular remodeling, inverse probability of treatment weighting

## Abstract

Total coronary revascularization via anterior thoracotomy (TCRAT) enables complete anatomical revascularization without sternotomy; however, data on its impact on left ventricular function remain limited. This study compared left ventricular functional outcomes between TCRAT and median sternotomy coronary artery bypass grafting (MS-CABG) in 554 patients undergoing elective isolated CABG at four centers (January 2020–January 2025) with preoperative and ≥3-month follow-up echocardiography. Patients were grouped as TCRAT (n = 241) or MS-CABG (n = 313). Stabilized inverse probability of treatment weighting was applied to reduce selection bias, achieving adequate covariate balance (all standardized mean differences < 0.10). The primary endpoint was follow-up left ventricular ejection fraction, assessed using IPTW-weighted analysis of covariance adjusted for preoperative values. No significant difference was observed between groups (β = 0.13; 95% CI, −0.63 to 0.90; *p* = 0.734). Adjusted left ventricular end-diastolic diameter was modestly higher in the MS-CABG group (β = 0.57 mm; 95% CI, 0.17–0.98; *p* = 0.006), while end-systolic diameter was similar. TCRAT was associated with longer operative times but shorter intensive care unit and hospital stays, along with lower transfusion requirements. These findings suggest that there was no statistically significant difference in follow-up systolic function between the surgical approaches. Although a modest difference in LVEDD was identified, its clinical significance remains uncertain.

## 1. Introduction

Coronary artery bypass grafting (CABG) remains the primary method for revascularizing patients with complex multivessel coronary artery disease, especially those with diabetes or impaired left ventricular function. Conventional median sternotomy coronary artery bypass grafting (MS-CABG) is the standard and most commonly used surgical approach for achieving complete revascularization [1,2,3,4]. Minimally invasive coronary surgery techniques have gained popularity due to their potential to reduce surgical trauma and promote faster recovery after surgery [5,6]. Total coronary revascularization via anterior thoracotomy (TCRAT) is a modern on-pump technique that allows for complete revascularization of multiple vessels without the need for sternotomy [7,8]. Early studies have shown its feasibility and safety in carefully selected patients [9,10]. However, despite these benefits, the effect of TCRAT on postoperative left ventricular systolic function and ventricular remodeling has not been well defined, especially when directly compared to conventional MS-CABG.

This study aimed to compare follow-up left ventricular systolic function, primarily measured by left ventricular ejection fraction (LVEF), and ventricular remodeling parameters between TCRAT and traditional MS-CABG.

## 2. Methods

### 2.1. Study Design and Patient Selection

**Figure 1:**
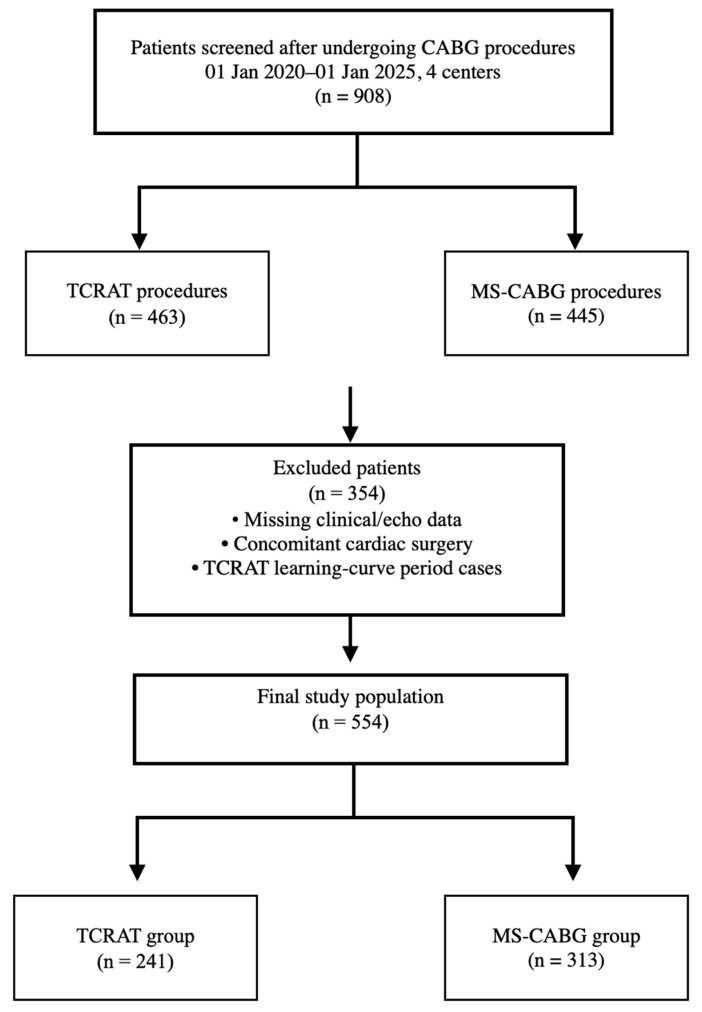
Study Flow Diagram of Patient Selection. A total of 908 patients who underwent CABG procedures at four centers between January 2020 and January 2025 were screened for eligibility. After applying predefined exclusion criteria, 554 patients were included in the final analysis (TCRAT, n = 241; MS-CABG, n = 313).

This study was designed as a multicenter retrospective cohort study. A total of 908 patients who underwent CABG procedures at four tertiary centers between 1 January 2020, and 1 January 2025, were initially screened for eligibility. Of these, 463 patients underwent TCRAT and 445 underwent MS-CABG. Only patients with an indication for isolated CABG and available transthoracic echocardiographic (TTE) data both preoperatively and at least 3 months after surgery were included. Left ventricular systolic function was assessed using left ventricular ejection fraction (LVEF). Patients with missing clinical or echocardiographic data, those undergoing concomitant cardiac procedures, emergency or redo surgery, concomitant valve or aortic surgery, estimated glomerular filtration rate < 30 mL/min/1.73 m^2^, or non-ischemic cardiomyopathy were excluded. In addition, to minimize the potential impact of the early learning curve, the first 30 consecutive TCRAT cases performed by each surgeon were excluded, although residual experience-related effects may still have persisted beyond this threshold. After applying these exclusion criteria, the final study population consisted of 554 patients, including 241 in the TCRAT group and 313 in the MS-CABG group. The patient selection process is summarized in Figure 1. To reduce variability related to surgical technique, only procedures performed during the same time period by experienced cardiovascular surgeons proficient in both techniques were included. Surgical techniques and perioperative care protocols were largely standardized across centers. The study was conducted in accordance with the Declaration of Helsinki [11] and approved by the Mersin University Clinical Research Ethics Committee (Approval No: 947; 3 September 2025). The approval was granted for the retrospective analysis of previously collected anonymized clinical data obtained during routine clinical care; no additional intervention, randomization, or prospective patient enrollment was performed for the purposes of this study.

### 2.2. Surgical Techniques

#### 2.2.1. Total Coronary Revascularization via Anterior Thoracotomy (TCRAT)

Patients were positioned supine with approximately 30° elevation of the left hemithorax. Double-lumen endotracheal intubation was used to enable single-lung ventilation, and intraoperative hemodynamic monitoring was routinely performed using transesophageal echocardiography (TEE). Femoral arterial and venous cannulation were routinely performed using standard techniques. Additional jugular venous cannulation was selectively used at the discretion of the operating surgeon in cases where enhanced venous drainage and right heart decompression were considered beneficial for operative exposure during multivessel revascularization through limited thoracotomy access. In male patients, the incision was made through the fourth intercostal space extending toward the sternocostal junction, whereas in female patients an inframammary approach was preferred. After entering the thoracic cavity, the pericardium was opened longitudinally. The left internal mammary artery (LIMA) was harvested in a skeletonized fashion, and saphenous vein grafts (SVG) were harvested endoscopically when indicated. Following initiation of cardiopulmonary bypass, the ascending aorta was cross-clamped, and myocardial protection was achieved using blood-based Del Nido cardioplegia. After completion of distal anastomoses, reperfusion was initiated with warm cardioplegia. Proximal anastomoses were generally performed using partial aortic clamping after careful mobilization and exposure of the ascending aorta through the thoracotomy. Adequate exposure was facilitated by patient positioning, pericardial traction sutures, and decompression under cardiopulmonary bypass. The specific proximal anastomotic strategy was individualized according to thoracic anatomy and intraoperative technical considerations. In the majority of TCRAT cases, revascularization was performed using a combination of LIMA and saphenous vein grafts, whereas total arterial revascularization was not routinely adopted during the study period.

#### 2.2.2. Median Sternotomy CABG (MS-CABG)

In the MS-CABG group, operations were performed via conventional median sternotomy. The LIMA was harvested in a pedicled fashion, and saphenous vein and/or radial artery grafts were obtained using standard techniques. Bilateral internal thoracic artery grafting was not routinely performed during the study period across the participating centers. Cardiopulmonary bypass was performed under normothermia, with arterial cannulation of the ascending aorta and venous cannulation performed according to surgeon preference and intraoperative requirements, most commonly through the right atrium, with bicaval cannulation used selectively when considered necessary.

Myocardial arrest was achieved using antegrade cold blood cardioplegia, which was repeated every 15–20 min. When feasible, the LIMA was preferentially anastomosed in situ to the left anterior descending artery (LAD), while other grafts were connected either directly to the ascending aorta or configured as T/Y composite grafts. Coronary anastomoses were performed using a continuous suture technique.

### 2.3. Data Collection and Definitions

All data were retrospectively obtained from hospital electronic records and operative reports. Preoperative variables included demographic characteristics (age, sex, body mass index), comorbidities (hypertension, diabetes mellitus, hyperlipidemia, chronic kidney disease), smoking status, prior myocardial infarction, EuroSCORE II, and medications (ACE inhibitors/ARBs, beta-blockers, and statins). Intraoperative variables included operative time, cardiopulmonary bypass and cross-clamp times, number and type of grafts, and intraoperative blood transfusion requirements. Postoperative variables included intensive care unit and hospital length of stay, as well as postoperative transfusion requirements. Complete revascularization was assessed using operative reports together with preoperative coronary angiographic findings and was defined anatomically as successful grafting of all major target coronary arteries with ≥70% stenosis and a vessel diameter ≥1.5 mm considered technically suitable for grafting by the operating surgeon. Postoperative angiographic confirmation of graft patency was not routinely available [12]. All these variables were included in the propensity score model as potential covariates associated with treatment selection and follow-up echocardiographic outcomes.

### 2.4. Echocardiographic Assessment and Study Endpoints

All echocardiographic measurements were obtained from routine clinical reports without centralized core laboratory adjudication. Because of the retrospective design, echocardiographers were not formally blinded to surgical approach, and interobserver variability could not be assessed. Preoperative transthoracic echocardiographic (TTE) measurements were performed within 7 days before surgery, whereas follow-up echocardiographic assessment was based on the first eligible TTE examination performed at least 3 months after surgery. Early evaluations (<3 months) were excluded, as measurements obtained before completion of early myocardial recovery may underestimate functional improvement and may be influenced by transient postoperative myocardial stunning [13]. LVEF was obtained from routine clinical transthoracic echocardiographic reports using the biplane Simpson method [14]. Reports not compliant with guideline-based measurements or containing incomplete data were excluded from the analysis.

#### 2.4.1. Primary Endpoint

The primary endpoint was follow-up LVEF (%). Although change in LVEF (ΔLVEF) may seem intuitive as a measure of treatment effect, change scores are inherently dependent on baseline values and susceptible to regression to the mean, which can cause systematic bias in group comparisons. Baseline-adjusted follow-up LVEF within an ANCOVA framework has been shown to provide a more statistically efficient and less biased estimate of treatment effect than change scores in studies with pre- and post-intervention measurements. The treatment effect between groups was assessed using IPTW-weighted linear regression models based on an ANCOVA framework, adjusted for preoperative LVEF. In these models, follow-up LVEF was the dependent variable, and preoperative LVEF was used as a covariate [15].

#### 2.4.2. Secondary Endpoints

Secondary clinical endpoints included operative time, cardiopulmonary bypass and cross-clamp durations, number of grafts, intraoperative red blood cell transfusion requirements, and lengths of stay in the intensive care unit and hospital. Additionally, follow-up left ventricular end-diastolic diameter (LVEDD) and left ventricular end-systolic diameter (LVESD), adjusted for their corresponding preoperative values, were analyzed as supportive indicators of ventricular remodeling.

#### 2.4.3. Predefined Subgroup Analysis

Patients were stratified into two predefined subgroups according to preoperative LVEF: preserved (≥50%) and reduced (<50%) [16]. This stratification was defined a priori to evaluate whether postoperative echocardiographic outcomes differed according to preoperative systolic function. Separate IPTW-weighted regression models were constructed within each subgroup.

### 2.5. Perioperative Management and Follow-Up

Perioperative care processes, including anesthesia protocols, cardiopulmonary bypass management, transfusion thresholds, and intensive care practices, were largely standardized across all centers. Early extubation, mobilization, and discharge criteria were implemented according to consistent institutional protocols. After discharge, all patients received guideline-directed medical therapy for coronary artery disease, which was continued according to clinical indication [3].

### 2.6. Statistical Analysis

Continuous variables are presented as mean (standard deviation) or median (interquartile range), as appropriate, and categorical variables as counts (percentages). Unadjusted comparisons were performed using appropriate parametric or nonparametric tests according to data distribution. To reduce confounding associated with the nonrandomized study design, inverse probability of treatment weighting (IPTW) was applied. Propensity scores were estimated using a multivariable logistic regression model including clinically relevant baseline variables. Stabilized weights targeting the average treatment effect (ATE) were used to reduce variance inflation associated with IPTW. The distribution of stabilized weights and propensity score overlap between groups were visually assessed and did not demonstrate substantial instability or severe extreme weights; therefore, weight trimming was not performed in order to preserve the target population and avoid unnecessary exclusion of observations. Covariate balance after weighting was evaluated using standardized mean differences (SMD), with values <0.10 considered indicative of adequate balance. Primary and secondary echocardiographic outcomes were analyzed using IPTW-weighted linear regression models within an ANCOVA framework. In each model, the corresponding follow-up echocardiographic parameter was treated as the dependent variable, and the respective baseline preoperative measurement was included as a covariate. This approach is recommended to account for baseline differences and minimize regression-to-the-mean effects [15]. β coefficients represent the average treatment effect (ATE) of MS-CABG relative to TCRAT and are reported with 95% confidence intervals. Robust (sandwich) standard errors were used to account for variance distortion introduced by weighting. Additional IPTW-weighted analyses of perioperative outcomes were performed as supportive descriptive analyses to improve consistency across study outcomes. Supplementary IPTW-weighted analyses evaluating change scores (Δ values) were also performed to provide complementary assessment of postoperative ventricular remodeling dynamics. Subgroup analyses according to predefined baseline LVEF categories were performed using the same IPTW-weighted modeling strategy. Corresponding supplementary analyses of change scores within LVEF subgroups were additionally conducted. Operative and perioperative outcomes were first summarized using unadjusted descriptive comparisons. In addition, IPTW-weighted analyses of continuous operative and recovery-related perioperative outcomes were performed as supportive outcome analyses to improve consistency with the primary echocardiographic analyses. Because these variables occurred after treatment allocation, they were not included in the propensity score estimation process. Standardized mean differences for operative and perioperative variables were additionally reported as descriptive measures of between-group effect size. All statistical analyses were performed using R software, version 4.4 (R Foundation for Statistical Computing, Vienna, Austria). A two-sided *p* value <0.05 was considered statistically significant. Given the retrospective design, no formal sample size calculation was performed. Analyses were conducted using complete-case data without multiple imputations.

## 3. Results

### 3.1. Baseline Characteristics

**Figure 2:**
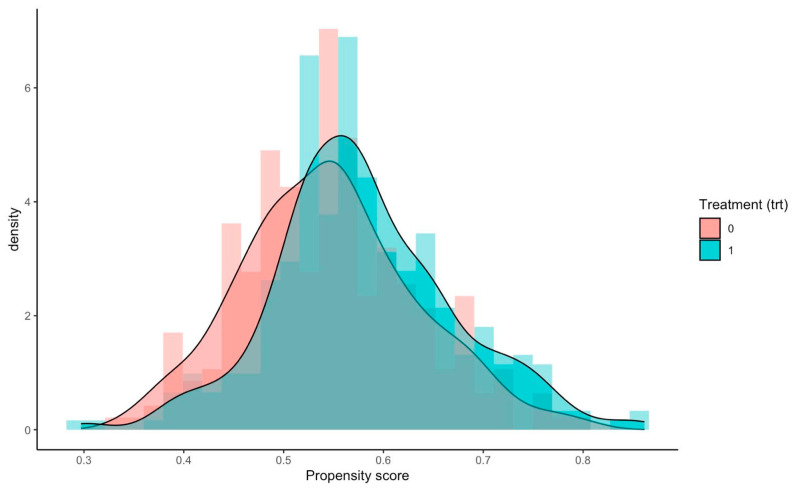
Distribution of propensity scores before and after IPTW adjustment. Distribution of propensity scores in the TCRAT and MS-CABG groups before and after IPTW adjustment. After weighting, the distributions demonstrated improved overlap between groups, indicating enhanced comparability of treatment groups.

**Figure 3:**
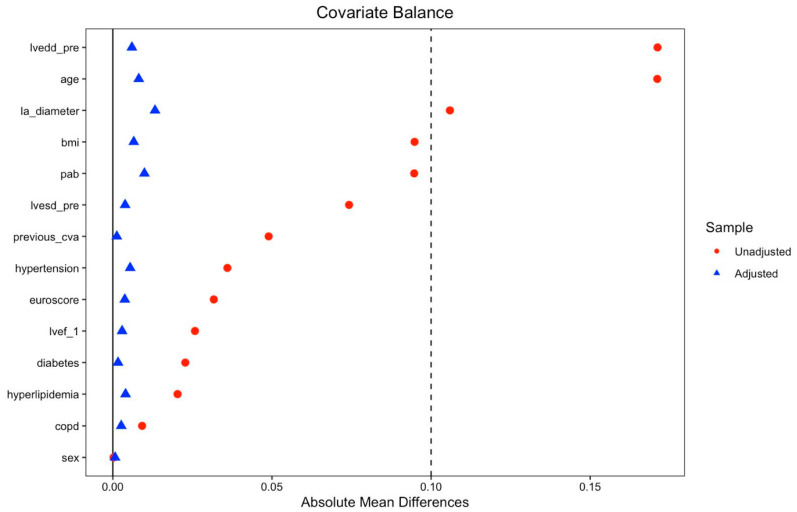
Covariate balance before and after IPTW adjustment (Love plot). Standardized mean differences (SMDs) of baseline covariates between the TCRAT and MS-CABG groups before (unadjusted) and after IPTW adjustment are shown. The vertical dashed line represents the predefined threshold for acceptable balance (SMD = 0.10). After weighting, all covariates were well balanced (SMD < 0.10), indicating successful adjustment for baseline differences.

After IPTW adjustment, adequate covariate balance was achieved between the TCRAT and MS-CABG groups across all baseline demographic and clinical variables, with all standardized mean differences (SMDs) below 0.10 (Table 1; Figure 2 and Figure 3). No meaningful differences were observed between the groups in weighted mean age, sex distribution, body mass index, preoperative ventricular dimensions, pulmonary artery pressure, EuroSCORE II, preoperative LVEF, or major comorbidities. Unadjusted baseline characteristics are presented in Supplementary Table S1.

### 3.2. Operative and Perioperative Findings

Operative and perioperative characteristics are summarized in Table 2A. The rates of complete revascularization were similar between the groups (TCRAT: 74.3% vs. MS-CABG: 76.4%; *p* = 0.560). Incomplete revascularization was most commonly related to diffuse distal disease, small-caliber target vessels, or technically unsuitable distal runoff identified intraoperatively. Operative time, cardiopulmonary bypass time, and cross-clamp time were significantly longer in the TCRAT group (all *p* < 0.001). In contrast, intensive care unit stay and total hospital stay were significantly shorter in the TCRAT group (*p* = 0.026 and *p* < 0.001, respectively). The proportion of patients requiring any blood transfusion was also significantly lower in the TCRAT group. In contrast, no significant differences were observed between groups regarding postoperative dialysis requirement, 30-day cerebrovascular events, surgical site infection, or 30-day mortality. The proportion of patients receiving ≥3 grafts was higher in the MS-CABG group (*p* = 0.002). Perioperative transfusion requirements were significantly lower in the TCRAT group (*p* < 0.001). Standardized mean differences for operative and perioperative variables are additionally presented in Supplementary Table S2 for descriptive interpretation of between-group effect sizes. IPTW-adjusted operative and perioperative outcome analyses are presented in Table 2B.

### 3.3. Echocardiographic Findings

#### 3.3.1. Unadjusted Analysis

Unadjusted echocardiographic data are shown in Table 3. Preoperative LVEF was similar between the groups (*p* = 0.601). No significant differences were seen in follow-up LVEF or ΔLVEF (*p* = 0.631 and *p* = 0.903, respectively). Follow-up LVEDD was notably lower in the TCRAT group (*p* = 0.002), while no significant difference was observed in ΔLVEDD (*p* = 0.094). No significant differences between groups were found for LVESD measurements. The interval from surgery to follow-up echocardiography was similar for both groups (TCRAT: 110.7 ± 16.9 days; MS-CABG: 108.3 ± 15.0 days; *p* = 0.082). Supplementary IPTW-adjusted analyses evaluating changes in echocardiographic parameters are presented in Supplementary Table S3. Consistent with the primary analyses, a modest difference was observed for ΔLVEDD, whereas no significant differences were identified for ΔLVESD or ΔLVEF.

#### 3.3.2. IPTW-Adjusted Analysis

In the IPTW-weighted regression analysis (Table 4), no significant difference was found between the surgical approaches with respect to the primary endpoint of follow-up LVEF (β = 0.13; 95% CI, −0.63 to 0.90; *p* = 0.734). In contrast, baseline-adjusted follow-up LVEDD was significantly higher in the MS-CABG group compared with the TCRAT group (β = 0.57; 95% CI, 0.17 to 0.98; *p* = 0.006). No significant difference was observed between the groups for follow-up LVESD (β = −0.03; 95% CI, −0.35 to 0.29; *p* = 0.856). The observed difference in LVEDD, although statistically significant, was modest in magnitude.

#### 3.3.3. Subgroup Analysis

In patients with preoperative LVEF <50%, no significant difference was observed between the groups in follow-up LVEF (*p* = 0.448). In contrast, follow-up LVEDD was significantly higher in the MS-CABG group compared with the TCRAT group (β = 2.16; 95% CI, 1.36 to 2.95; *p* < 0.001). In patients with preoperative LVEF ≥50%, no significant differences were observed between the groups in follow-up LVEF (*p* = 0.392) or follow-up LVEDD (*p* = 0.751). However, follow-up LVESD was modestly lower in the MS-CABG group (β = −0.28; 95% CI, −0.50 to −0.07; *p* = 0.011). No formal interaction testing was performed, and the study was not powered for subgroup-specific comparisons; therefore, subgroup findings should be interpreted as exploratory and hypothesis-generating. These results are presented in Table 5. Supplementary IPTW-adjusted analyses evaluating changes in echocardiographic parameters in patients with reduced baseline LVEF are additionally presented in Supplementary Table S4. Consistent with the primary subgroup analyses, a greater reduction in ΔLVEDD was observed in patients with reduced baseline LVEF, whereas no significant between-group differences were identified for ΔLVESD or ΔLVEF.

## 4. Discussion

In this multicenter study, no significant difference was observed in follow-up LVEF adjusted for preoperative values between the TCRAT and MS-CABG groups. In contrast, follow-up LVEDD was significantly higher in the MS-CABG group compared with TCRAT, whereas no significant difference was observed in follow-up LVESD. Although the LVEDD difference appeared numerically larger among patients with reduced preoperative LVEF, these subgroup findings should be interpreted cautiously because no formal interaction testing was performed and the analyses were exploratory in nature. However, given the observational nature of the study and the possibility of unmeasured treatment-selection factors, these findings should not be interpreted as demonstrating a causal effect of TCRAT on ventricular remodeling.

The similar rates of anatomically defined complete revascularization between the groups suggest that both surgical strategies achieved broadly comparable revascularization goals at the operative level. However, the lower frequency of ≥3-vessel grafting in the TCRAT group raises the possibility that patients selected for the minimally invasive approach may have had less extensive or technically less complex coronary disease. Because detailed angiographic complexity variables and target-vessel burden were not systematically available, residual selection bias related to coronary anatomy cannot be excluded. Accordingly, when effective revascularization is achieved, postoperative left ventricular systolic recovery may be largely influenced by myocardial perfusion adequacy. Exploratory subgroup analyses showed generally consistent findings across baseline LVEF strata. Although these observations should be interpreted cautiously, given the exploratory nature of the subgroup analyses and the potential for type I error from multiple comparisons. However, detailed angiographic characteristics and myocardial substrate-related variables, including coronary anatomical complexity, diffuse disease burden, chronic total occlusions, left main involvement, and myocardial scar burden, were not systematically available. These factors may substantially influence postoperative ventricular recovery and may have contributed to the absence of an observed difference in follow-up LVEF between groups. Moreover, the complete revascularization rates observed in the present study were lower than those reported in some highly selected surgical series, likely reflecting the inclusion of real-world patients with diffuse multivessel disease and variable distal target quality. These anatomical factors may also have influenced postoperative ventricular remodeling outcomes.

Recovery of left ventricular systolic function after coronary revascularization is a heterogeneous process, and a substantial proportion of patients with ischemic cardiomyopathy may not exhibit a meaningful increase in LVEF [17,18]. Previous studies have demonstrated that postoperative functional improvement is determined not solely by the surgical technique, but by a complex interplay of patient- and disease-related factors, including the severity of preoperative ventricular dysfunction, the presence of myocardial viability, and the completeness of revascularization [17,19,20]. In particular, long-term analyses of the STICH trial have shown that restoration of blood flow to viable myocardium is a key determinant of left ventricular functional recovery [1]. Similarly, viability-focused studies have demonstrated that revascularization can provide meaningful functional benefit in the presence of dysfunctional but viable myocardium [19]. However, more recent randomized data suggest that the impact of revascularization on left ventricular function may be more limited than previously assumed and highly dependent on underlying myocardial substrate characteristics. In this context, the REVIVED-BCIS2 trial reported that the addition of percutaneous revascularization to optimal medical therapy did not improve LVEF or major clinical outcomes in patients with severe left ventricular dysfunction [21]. Accordingly, the similar LVEF recovery observed between TCRAT and MS-CABG in our study is biologically plausible. Comparable rates of revascularization were achieved in both groups, and restoration of myocardial perfusion under cardiopulmonary bypass and cardioplegic arrest represents a shared physiological mechanism. Taken together, these findings support the interpretation that myocardial viability and baseline disease characteristics may play an important role in determining systolic functional recovery. Consistent with our subgroup findings, the absence of a differential effect of surgical approach on follow-up LVEF across preoperative LVEF strata further corroborates this mechanistic framework.

Although LVEF recovery was similar between the two surgical approaches, a modest difference in follow-up LVEDD was observed. However, the magnitude of this difference was small, and its clinical relevance remains uncertain, particularly in the absence of corresponding differences in follow-up LVEF or LVESD. Remodeling and systolic functional recovery after revascularization do not always progress in parallel, and early geometric changes may not necessarily translate into measurable functional differences [18]. In addition, the echocardiographic follow-up period in the present study primarily reflects early postoperative remodeling rather than stable long-term ventricular adaptation [22,23,24]. Therefore, the observed LVEDD finding should be interpreted cautiously and considered primarily exploratory and hypothesis-generating rather than indicative of a clinically meaningful remodeling benefit associated with the surgical approach.

From a perioperative perspective, TCRAT was associated with longer operative, cardiopulmonary bypass, and cross-clamp times, likely reflecting the increased technical complexity and more limited surgical exposure inherent to this approach. Nevertheless, intensive care unit and hospital length of stay were shorter, and transfusion requirements were lower in the TCRAT group. Previous studies have similarly reported that minimally invasive coronary surgery may facilitate faster postoperative recovery despite greater intraoperative technical demands [10,25,26,27]. However, multiple competing perioperative factors may influence postoperative recovery and ventricular remodeling, including surgical trauma, extent of pericardial opening, cardiopulmonary bypass exposure, myocardial protection strategy, and cross-clamp duration. In particular, although minimally invasive access may theoretically reduce surgical trauma and pericardial disruption, all patients in the TCRAT group underwent on-pump surgery with aortic cross-clamping, and both cardiopulmonary bypass and cross-clamp times were longer than in the MS-CABG group, which may themselves affect postoperative outcomes. Residual learning-curve effects associated with advanced minimally invasive coronary surgery may also have contributed to the longer operative and cardiopulmonary bypass times observed in the TCRAT group. In addition, graft-selection strategies were not fully standardized across centers, and total arterial revascularization was not routinely performed, which may limit generalizability to centers that preferentially use multi-arterial grafting approaches.

From a clinical perspective, the absence of a difference in LVEF between the two approaches suggests that surgical access alone may not be a primary determinant of systolic functional recovery when complete revascularization is achieved. Therefore, the choice of surgical technique may be guided more by perioperative considerations and patient-specific factors rather than expectations of differential impact on left ventricular systolic function.

This study has several limitations. First, owing to the retrospective observational design, residual and unmeasured confounding cannot be excluded despite IPTW-based adjustment. Although measured baseline variables were well balanced after weighting, treatment selection for TCRAT versus MS-CABG may still have been influenced by factors not captured in the propensity score model, including coronary anatomical complexity, distal target quality, frailty, surgeon preference, center-specific practice patterns, and aortic pathology. In particular, detailed angiographic and myocardial substrate-related variables, such as SYNTAX score, diffuse coronary disease burden, chronic total occlusions, left main disease, myocardial scar burden, and target-vessel burden, were not consistently available across centers and therefore could not be incorporated into the analysis. Since these factors may influence both treatment selection and postoperative ventricular recovery, residual confounding related to coronary disease severity cannot be excluded. Accordingly, the observed differences in LVEDD should be interpreted as associative and hypothesis-generating rather than evidence of a causal effect of surgical approach. Second, the present study primarily evaluated echocardiographic surrogate parameters rather than long-term clinical outcomes. Although additional perioperative clinical outcomes were assessed, comprehensive long-term outcome data—including survival, major adverse cardiovascular events, myocardial infarction, stroke, repeat revascularization, graft patency, and heart failure progression—were not systematically available. Therefore, the prognostic and clinical significance of the modest structural differences observed in ventricular dimensions remains uncertain. Third, echocardiographic measurements were obtained from routine clinical reports without centralized core-laboratory validation, and echocardiographers were not formally blinded to surgical approach. Moreover, the echocardiographic follow-up period was relatively short and primarily reflects early postoperative remodeling rather than stable long-term ventricular adaptation. Ventricular remodeling after coronary revascularization may continue for substantially longer periods, particularly in patients with ischemic cardiomyopathy or impaired baseline ventricular function. In addition, systematic myocardial viability assessment was not available, limiting the ability to fully interpret the relationship between revascularization and postoperative functional recovery. Fourth, although early cases were excluded to reduce learning-curve effects, the learning process associated with advanced minimally invasive coronary surgery may extend beyond the first 30 procedures. Therefore, residual operator experience and center-related heterogeneity cannot be fully excluded and may have influenced operative efficiency and perioperative outcomes. In addition, graft-selection strategies—including LIMA utilization and multiarterial grafting—were not fully standardized across centers, reflecting real-world surgical decision-making and institutional practice variation. Similarly, intraoperative graft assessment using transit-time flow measurement was neither standardized nor consistently available, limiting evaluation of graft quality and procedural uniformity. Finally, subgroup analyses were exploratory and performed without formal interaction testing, increasing the possibility of type I error due to multiple comparisons. Nevertheless, the multicenter design, the relatively large cohort, the largely standardized surgical practices, and the achievement of adequate covariate balance after IPTW adjustment strengthen the interpretability of the findings. In addition, assessment of left ventricular function at least 3 months after surgery reduces the potential impact of transient early postoperative myocardial dysfunction on the results. Future prospective studies incorporating comprehensive angiographic characterization, myocardial viability assessment, long-term clinical outcomes, and graft-patency evaluation are needed to clarify the clinical relevance of the observed echocardiographic findings.

## 5. Conclusions

In this multicenter study, TCRAT and MS-CABG were associated with similar follow-up left ventricular systolic function. Early follow-up LVEDD was modestly lower in the TCRAT group. Although operative times were longer with TCRAT, intensive care unit and hospital length of stay were shorter and transfusion requirements were lower. These findings suggest that, when comparable revascularization is achieved, TCRAT appears to be a feasible minimally invasive alternative to MS-CABG in selected patients.

## Figures and Tables

**Table 1:** Baseline Characteristics After IPTW Adjustment (ATE-Weighted Sample).

Variable	TCRAT (Weighted)	MS-CABG (Weighted)	SMD
Effective sample size (n)	241.3	312.9	—
Age, years	64.66 ± 9.71	64.60 ± 11.51	0.005
Male sex, n (%)	181.5 (75.2)	235.6 (75.2)	<0.001
Body mass index, kg/m^2^	28.62 ± 3.30	28.59 ± 3.21	0.008
LVEDD (preoperative), mm	49.55 ± 4.21	49.52 ± 4.68	0.006
LVESD (preoperative), mm	33.92 ± 4.41	33.86 ± 5.47	0.012
Left atrial diameter, mm	37.04 ± 5.31	36.96 ± 4.32	0.015
Pulmonary artery pressure, mmHg	28.84 ± 8.46	28.77 ± 7.96	0.009
EuroSCORE II, %	4.32 ± 1.95	4.33 ± 2.16	0.004
Preoperative LVEF, %	50.21 ± 8.37	50.33 ± 8.18	0.014
COPD, n (%)	66.3 (27.5)	85.1 (27.2)	0.007
Hyperlipidemia, n (%)	97.4 (40.4)	128.5 (41.0)	0.014
Hypertension, n (%)	139.6 (57.8)	179.7 (57.4)	0.009
Diabetes mellitus, n (%)	103.6 (42.9)	133.2 (42.5)	0.008
History of cerebrovascular event, n (%)	25.5 (10.6)	33.3 (10.6)	0.002

Values are presented as IPTW-weighted mean ± standard deviation or weighted count (%), unless otherwise indicated. IPTW with stabilized weights targeting the average treatment effect (ATE) was applied. Covariate balance was assessed using standardized mean differences (SMD), with values <0.10 indicating adequate balance. No hypothesis testing was performed for baseline comparisons. Effective sample size represents the sum of stabilized weights within each group. MS-CABG, median sternotomy coronary artery bypass grafting; TCRAT, total coronary revascularization via anterior thoracotomy, ATE, average treatment effect; COPD, chronic obstructive pulmonary disease; IPTW, inverse probability of treatment weighting; LVEF, left ventricular ejection fraction; LVEDD, left ventricular end-diastolic diameter; LVESD, left ventricular end-systolic diameter; SMD, standardized mean difference.

**Table 2:** (**A**). Operative and Perioperative Outcomes (Unadjusted). (**B**). IPTW-Adjusted Operative and Perioperative Outcomes.

(**A**)			
**Variable**	**TCRAT** **(n = 241)**	**MS-CABG** **(n = 313)**	***p*-Value**
Operation time, min	270.00 ± 68.39	211.78 ± 53.78	<0.001
Cardiopulmonary bypass time, min	152.22 ± 44.86	108.11 ± 32.61	<0.001
Cross-clamp time, min	82.22 ± 28.87	59.74 ± 19.83	<0.001
Complete revascularization, n (%)	179 (74.3)	239 (76.4)	0.560
Number of grafts, n (%)			0.002
1 graft	33 (14.0)	23 (7.3)	
2 grafts	46 (19.1)	50 (16.0)	
≥3 grafts	162 (67.2)	240 (76.7)	
LIMA use, n (%)	205 (85.1)	284 (90.7)	0.054
ICU stay, hours	41.57 ± 25.64	47.09 ± 32.44	0.026
Hospital stay, days	5.65 ± 1.55	6.22 ± 1.54	<0.001
Perioperative blood transfusion, n (%)			<0.001
0 units	32 (13.3)	2 (0.6)	
1 unit	71 (29.5)	31 (9.9)	
2 units	75 (31.1)	93 (29.7)	
≥3 units	63 (26.1)	187 (59.7)	
Any blood transfusion, n (%)	209 (86.7)	311 (99.4)	<0.001
Postoperative dialysis, n (%)	5 (2.1)	7 (2.2)	1.000 *
30-day cerebrovascular event, n (%)	7 (2.9)	8 (2.6)	0.799 *
Infection, n (%)	6 (2.5)	12 (3.8)	0.472 *
30-day mortality, n (%)	0 (0.0)	0 (0.0)	-
(**B**)			
**Variable**	**Adjusted** **Difference**	**95% CI**	***p*-Value**
Operation time, min	+57.86	47.69 to 68.04	<0.001
Cardiopulmonary bypass time, min	+44.23	37.78 to 50.67	<0.001
Cross-clamp time, min	+22.75	18.65 to 26.84	<0.001
ICU stay, hours	−3.41	−8.38 to 1.56	0.178
Hospital stay, days	−0.48	−0.74 to −0.21	<0.001

Values are presented as mean ± standard deviation or n (%), unless otherwise indicated. *p*-values are derived from unadjusted between-group comparisons and are provided for descriptive purposes only. Operative and perioperative variables were not included in the propensity score model, as they occur after treatment allocation. Categorical variables were compared using the chi-square test. Transfusion categories reflect the total number of units administered during the perioperative period. Abbreviations: MS-CABG, median sternotomy coronary artery bypass grafting; TCRAT, total coronary revascularization via anterior thoracotomy, ICU, intensive care unit; LIMA, left internal mammary artery. Footnote: For Table 2B, analyses were performed using IPTW-adjusted weighted linear regression models. Positive values indicate higher values in the TCRAT group relative to the MS-CABG group, whereas negative values indicate lower values in the TCRAT group. Operative and perioperative variables were not included in the propensity score estimation process because they occurred after treatment allocation. * Fisher’s exact test was used because of low event counts.

**Table 3:** Unadjusted Echocardiographic Outcomes and Changes From Baseline.

Parameter	TCRAT (n = 241)	MS-CABG (n = 313)	*p*-Value
LVEF, %			
Preoperative	50.60 ± 7.65	50.23 ± 8.23	0.601
Follow-up	52.90 ± 10.25	52.59 ± 8.01	0.631
ΔLVEF (follow-up − preoperative)	2.30 ± 4.73	2.35 ± 5.14	0.903
LVEDD, mm			
Preoperative	49.09 ± 3.96	49.83 ± 4.73	0.049
Follow-up	47.95 ± 3.39	49.05 ± 4.70	0.002
ΔLVEDD (follow-up − preoperative)	−1.13 ± 2.67	−0.78 ± 2.28	0.094
LVESD, mm			
Preoperative	33.60 ± 4.14	33.96 ± 5.57	0.395
Follow-up	32.98 ± 4.37	33.33 ± 5.34	0.415
ΔLVESD (follow-up − preoperative)	−0.61 ± 2.62	−0.63 ± 1.21	0.912
Follow-up interval, days	110.7 ± 16.9	108.3 ± 15.0	0.082
Left atrial diameter, mm	36.63 ± 5.15	37.14 ± 4.37	0.222
Pulmonary artery pressure, mmHg	28.37 ± 8.20	29.13 ± 7.97	0.271

Values are presented as mean ± standard deviation. Unweighted sample sizes are shown. *p*-values are provided for descriptive purposes only and should not be interpreted as evidence of group differences due to the non-randomized study design. Δ values represent the change from baseline to follow-up (follow-up minus preoperative). Follow-up echocardiographic measurements were obtained at the first eligible transthoracic echocardiographic examination performed ≥3 months after surgery. Follow-up interval represents the time from surgery to this assessment. Abbreviations: MS-CABG, median sternotomy coronary artery bypass grafting; TCRAT, total coronary revascularization via anterior thoracotomy; LVEF, left ventricular ejection fraction; LVEDD, left ventricular end-diastolic diameter; LVESD, left ventricular end-systolic diameter.

**Table 4:** IPTW-Weighted Analysis of Echocardiographic Outcomes.

Outcome	TCRAT (Weighted Mean ± SD)	MS-CABG (Weighted Mean ± SD)	β Coefficient (95% CI)	*p*-Value
Follow-up LVEF (%)	52.52 ± 7.44	52.74 ± 7.88	0.13 (−0.63 to 0.90)	0.734
Follow-up LVEDD (mm)	48.21 ± 3.48	48.76 ± 4.60	0.57 (0.17 to 0.98)	0.006
Follow-up LVESD (mm)	33.31 ± 4.44	33.22 ± 5.23	−0.03 (−0.35 to 0.29)	0.856

Values are presented as IPTW-weighted mean ± standard deviation. Analyses were performed using IPTW-weighted linear regression models adjusted for the corresponding preoperative echocardiographic value. β coefficients represent adjusted differences between groups (MS-CABG minus TCRAT) and correspond to the average treatment effect (ATE). Stabilized IPTW weights targeting the ATE were applied. Two-sided *p*-values < 0.05 were considered statistically significant. Abbreviations: ATE, average treatment effect; CI, confidence interval; IPTW, inverse probability of treatment weighting; LVEF, left ventricular ejection fraction; LVEDD, left ventricular end-diastolic diameter; LVESD, left ventricular end-systolic diameter; MS-CABG, median sternotomy coronary artery bypass grafting; TCRAT, total coronary revascularization via anterior thoracotomy.

**Table 5:** IPTW-Weighted Analysis of Echocardiographic Outcomes Stratified by LVEF.

Subgroup	Outcome	TCRAT (Weighted Mean ± SD)	MS-CABG (Weighted Mean ± SD)	β Coefficient (95% CI)	*p*-Value
Reduced LVEF (<50%) (n = 164)	Follow-up LVEF (%)	45.15 ± 7.16	44.45 ± 7.46	−0.58 (−2.06 to 0.91)	0.448
	Follow-up LVEDD (mm)	50.35 ± 3.75	53.43 ± 3.69	2.16 (1.36 to 2.95)	<0.001
	Follow-up LVESD (mm)	36.23 ± 5.28	38.87 ± 4.79	0.62 (−0.46 to 1.69)	0.261
Preserved LVEF (≥50%) (n = 390)	Follow-up LVEF (%)	56.00 ± 4.42	56.14 ± 5.01	0.37 (−0.47 to 1.20)	0.392
	Follow-up LVEDD (mm)	47.20 ± 2.84	46.85 ± 3.42	−0.07 (−0.53 to 0.38)	0.751
	Follow-up LVESD (mm)	31.93 ± 3.18	30.90 ± 3.26	−0.28 (−0.50 to −0.07)	0.011

Values are presented as IPTW-weighted mean ± standard deviation. Analyses were performed using IPTW-weighted linear regression models adjusted for the corresponding preoperative echocardiographic value within each preoperative LVEF stratum. β coefficients represent adjusted differences between groups (MS-CABG minus TCRAT) and correspond to the average treatment effect (ATE). Stabilized IPTW weights targeting the ATE were applied. Two-sided *p*-values <0.05 were considered statistically significant. Abbreviations: ATE, average treatment effect; CI, confidence interval; IPTW, inverse probability of treatment weighting; LVEF, left ventricular ejection fraction; LVEDD, left ventricular end-diastolic diameter; LVESD, left ventricular end-systolic diameter; MS-CABG, median sternotomy coronary artery bypass grafting; TCRAT, total coronary revascularization via anterior thoracotomy.

## Data Availability

The datasets generated and/or analyzed during the current study are available from the corresponding author upon reasonable request.

## Supplementary Materials

The following supporting information can be downloaded at: https://www.mdpi.com/article/10.3390/jcdd13060244/s1, Table S1: Baseline Characteristics Before IPTW Adjustment (Unweighted Sample); Table S2: Standardized Mean Differences for Operative and Perioperative Variables; Table S3: IPTW-Adjusted Changes in Echocardiographic Parameters; Table S4: IPTW-Adjusted Changes in Echocardiographic Parameters in Patients With Reduced Baseline LVEF.
