# Supplementary material for: Left Ventricular Remodeling After Total Coronary Revascularization via Anterior Thoracotomy Versus Conventional Coronary Artery Bypass Grafting
Source: J Cardiovasc Dev Dis. 2026 Jun 3;13(6):244. doi: 10.3390/jcdd13060244 (PMC13301010; doi:10.3390/jcdd13060244)
Supplement: Supplementary file 1 [file jcdd-13-00244-s001.zip › Supplementary Table S1.pdf]

Supplementary Table S1. Baseline Characteristics Before IPTW Adjustment (Unweighted Sample)

Variable	TCRAT (n = 241)	MS-CABG (n = 313)	p-value
**Age, years**	63.63 ± 9.80	65.44 ± 11.30	0.048
**Male sex, n (%)**	181 (75.1)	235 (75.1)	0.999
**Body mass index, kg/m²**	28.44 ± 3.24	28.74 ± 3.23	0.269
**COPD, n (%)**	64 (26.6)	86 (27.5)	0.885
**Hyperlipidemia, n (%)**	105 (43.6)	130 (41.5)	0.694
**Hypertension, n (%)**	133 (55.2)	184 (58.8)	0.446
**Diabetes mellitus, n (%)**	100 (41.5)	137 (43.8)	0.653
**History of cerebrovascular event, n (%)**	19 (7.9)	40 (12.8)	0.087
**EuroSCORE II, %**	4.29 ± 1.93	4.35 ± 2.17	0.713
**Preoperative LVEF, %**	50.60 ± 8.21	50.23 ± 8.23	0.601
**LVEDD (preoperative), mm**	49.09 ± 3.96	49.83 ± 4.73	0.049
**LVESD (preoperative), mm**	33.60 ± 4.14	33.96 ± 5.57	0.395
**Left atrial diameter, mm**	36.63 ± 5.15	37.14 ± 4.37	0.222
**Pulmonary artery pressure, mmHg**	28.37 ± 8.20	29.13 ± 7.97	0.271

Abbreviations: COPD, chronic obstructive pulmonary disease; LVEF, left ventricular ejection fraction; LVEDD, left ventricular end-diastolic diameter; LVESD, left ventricular end-systolic diameter; MS-CABG, median sternotomy coronary artery bypass grafting; TCRAT, total coronary revascularization via anterior thoracotomy. **Footnote:** Values are presented as mean ± standard deviation or n (%), unless otherwise indicated. p-values are derived from unadjusted between-group comparisons and are provided for descriptive purposes only. These comparisons do not account for baseline imbalances or treatment selection bias and should not be interpreted as evidence of causal differences between groups.
