# Supplementary material for: Left Ventricular Remodeling After Total Coronary Revascularization via Anterior Thoracotomy Versus Conventional Coronary Artery Bypass Grafting
Source: J Cardiovasc Dev Dis. 2026 Jun 3;13(6):244. doi: 10.3390/jcdd13060244 (PMC13301010; doi:10.3390/jcdd13060244)
Supplement: Supplementary file 1 [file jcdd-13-00244-s001.zip › Supplementary Table S2.pdf]

Supplementary Table S2. Standardized Mean Differences for Operative and Perioperative Variables

Variable	TCRAT (n = 241)	MS-CABG (n = 313)	SMD
**Operation time, min**	270.00 ± 68.39	211.78 ± 53.78	0.94
**Cardiopulmonary bypass time, min**	152.22 ± 44.86	108.11 ± 32.61	1.12
**Cross-clamp time, min**	82.22 ± 28.87	59.74 ± 19.83	0.91
**ICU stay, hours**	41.57 ± 25.64	47.09 ± 32.44	0.19
**Hospital stay, days**	5.65 ± 1.55	6.22 ± 1.54	0.37

Abbreviations: MS-CABG, median sternotomy coronary artery bypass grafting; TCRAT, total coronary revascularization via anterior thoracotomy, ICU, intensive care unit

Footnote: Values are presented as mean ± standard deviation. Standardized mean differences are presented for descriptive purposes only. Operative and perioperative variables were not included in the propensity score model because they occurred after treatment allocation; therefore, these SMDs should not be interpreted as measures of baseline covariate balance.
