# Supplementary material for: Left Ventricular Remodeling After Total Coronary Revascularization via Anterior Thoracotomy Versus Conventional Coronary Artery Bypass Grafting
Source: J Cardiovasc Dev Dis. 2026 Jun 3;13(6):244. doi: 10.3390/jcdd13060244 (PMC13301010; doi:10.3390/jcdd13060244)
Supplement: Supplementary file 1 [file jcdd-13-00244-s001.zip › Supplementary Table S3.pdf]

Supplementary Table S3. IPTW-Adjusted Changes in Echocardiographic Parameters

Variable	Adjusted Difference	95% CI	p-value
**ΔLVEDD, mm**	-0.59	−1.01 to −0.16	0.007
**ΔLVESD, mm**	+0.02	−0.29 to 0.34	0.881
**ΔLVEF, %**	-0.10	−0.95 to 0.74	0.809

Abbreviations: IPTW, inverse probability of treatment weighting; LVEDD, left ventricular end-diastolic diameter; LVEF, left ventricular ejection fraction; LVESD, left ventricular end-systolic diameter

Footnote: Analyses were performed using IPTW-adjusted weighted linear regression models. Negative values indicate greater reductions in ventricular dimensions in the TCRAT group relative to the MS-CABG group.
