# Supplementary material for: Left Ventricular Remodeling After Total Coronary Revascularization via Anterior Thoracotomy Versus Conventional Coronary Artery Bypass Grafting
Source: J Cardiovasc Dev Dis. 2026 Jun 3;13(6):244. doi: 10.3390/jcdd13060244 (PMC13301010; doi:10.3390/jcdd13060244)
Supplement: Supplementary file 1 [file jcdd-13-00244-s001.zip › Supplementary Table S4.pdf]

Supplementary Table S4. IPTW-Adjusted Changes in Echocardiographic Parameters in Patients With Reduced Baseline LVEF

Variable	Adjusted Difference	95% CI	p-value
**ΔLVEDD, mm**	-1.75	−2.60 to −0.91	<0.001
**ΔLVESD, mm**	-0.36	−1.25 to 0.53	0.425
**ΔLVEF, %**	+0.56	−0.89 to 2.01	0.445

Abbreviations: IPTW, inverse probability of treatment weighting; LVEDD, left ventricular end-diastolic diameter; LVEF, left ventricular ejection fraction; LVESD, left ventricular end-systolic diameter

Footnote: Analyses were performed using IPTW-adjusted weighted linear regression models in patients with reduced baseline LVEF. Negative values indicate greater reductions in ventricular dimensions in the TCRAT group relative to the MS-CABG group.
